# Supplementary material for: The ER folding sensor UGGT1 acts on TAPBPR-chaperoned peptide-free MHC I
Source: eLife. 2023 Jun 22;12:e85432. doi: 10.7554/eLife.85432 (PMC10325711; doi:10.7554/eLife.85432)

Figure 1—source data 1

Original unedited SDS-PAGE gel of HLA-A*68:02$^{Fos}$-TAPBPR$^{Jun}$, Figure 1C

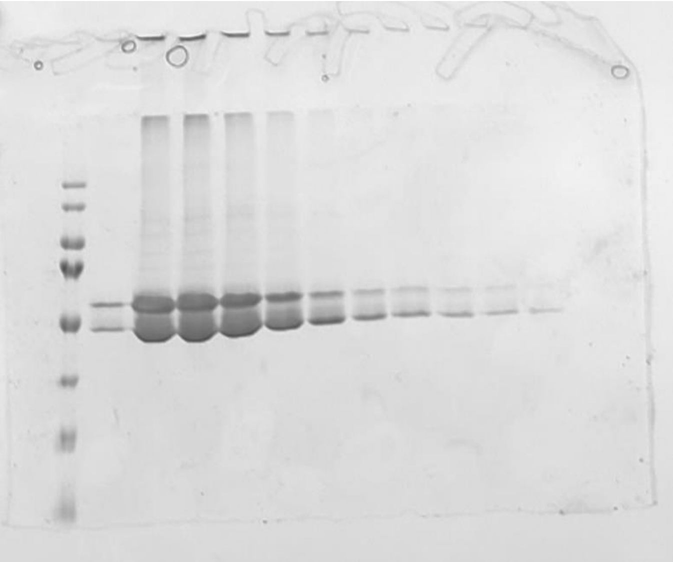

Original uncropped SDS-PAGE gel of HLA-A*68:02$^{Fos}$-TAPBPR$^{Jun}$ with highlighted relevant bands, Figure 1C

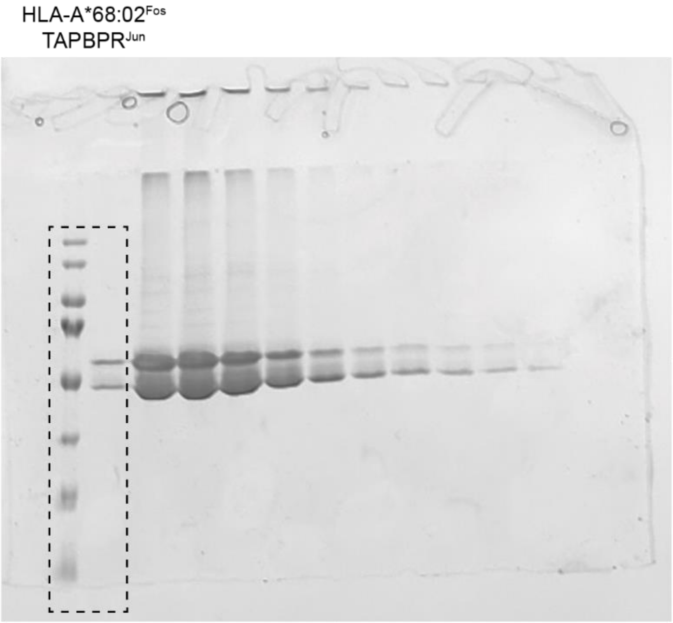

Supplement: Figure 1—source data 1. [file elife-85432-fig1-data1.zip › Figure 1-source data 1/Figure 1-source data 1.pdf]
