# Supplementary material for: The ER folding sensor UGGT1 acts on TAPBPR-chaperoned peptide-free MHC I
Source: eLife. 2023 Jun 22;12:e85432. doi: 10.7554/eLife.85432 (PMC10325711; doi:10.7554/eLife.85432)

Figure 1—figure supplement 2—source data 2

Original unedited SDS-PAGE gel of UGGT1$^{D1316N}$, Figure 1—figure supplement 2C

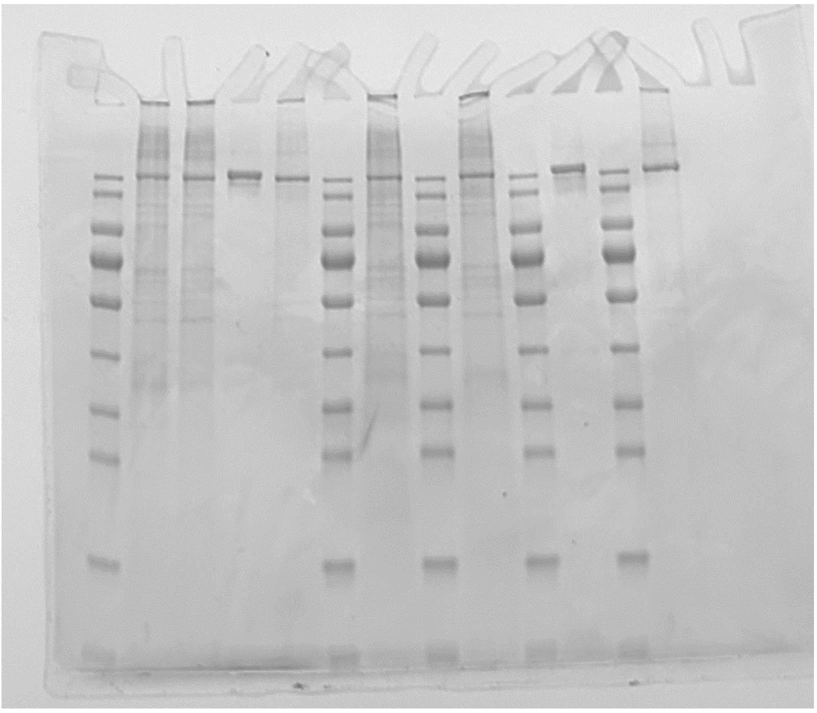

Original uncropped SDS-PAGE gel of UGGT1$^{D1316N}$ with highlighted relevant bands, Figure 1—figure supplement 2C

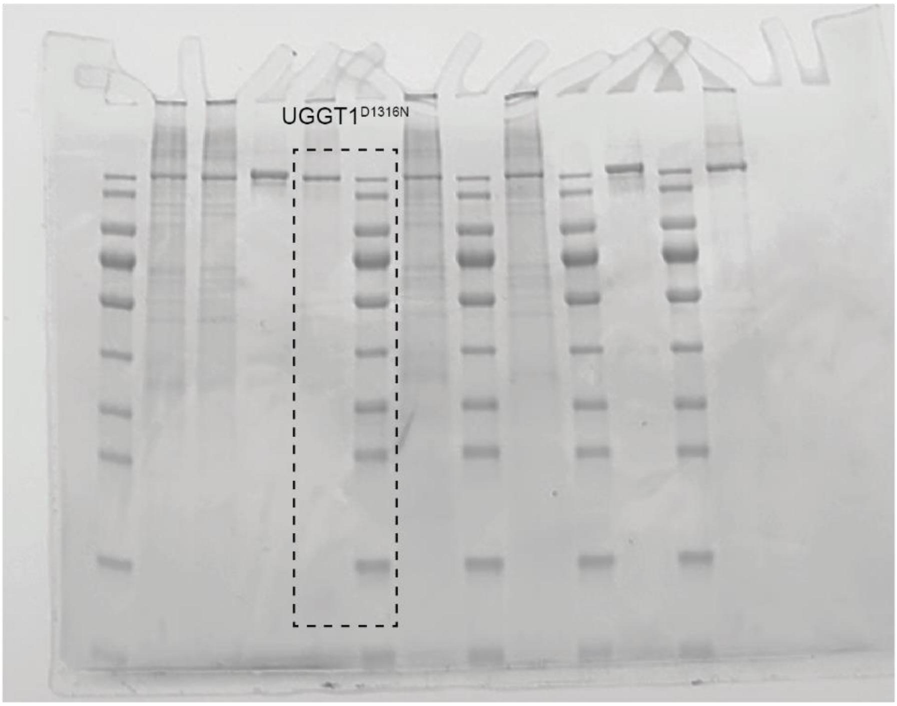

Supplement: Figure 1—figure supplement 2—source data 2. [file elife-85432-fig1-figsupp2-data2.zip › Figure 1-figure supplement 2-source data 2/Figure 1-figure supplement 2-source data 2.pdf]
