# Supplementary material for: The ER folding sensor UGGT1 acts on TAPBPR-chaperoned peptide-free MHC I
Source: eLife. 2023 Jun 22;12:e85432. doi: 10.7554/eLife.85432 (PMC10325711; doi:10.7554/eLife.85432)

Figure 4—source data 3

Original unedited SDS-PAGE gel of pull-down experiment to test $Ca^{2+}$ dependence of $UGGT1^{wt}$, Figure 4F

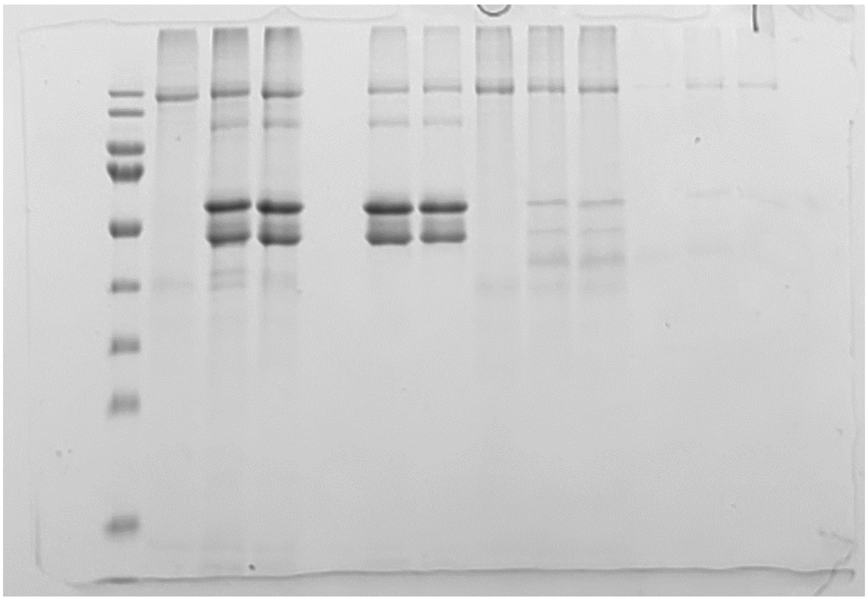

Original uncropped SDS-PAGE gel of pull-down experiment to test $Ca^{2+}$ dependence of $UGGT1^{wt}$ with highlighted relevant bands, Figure 4F

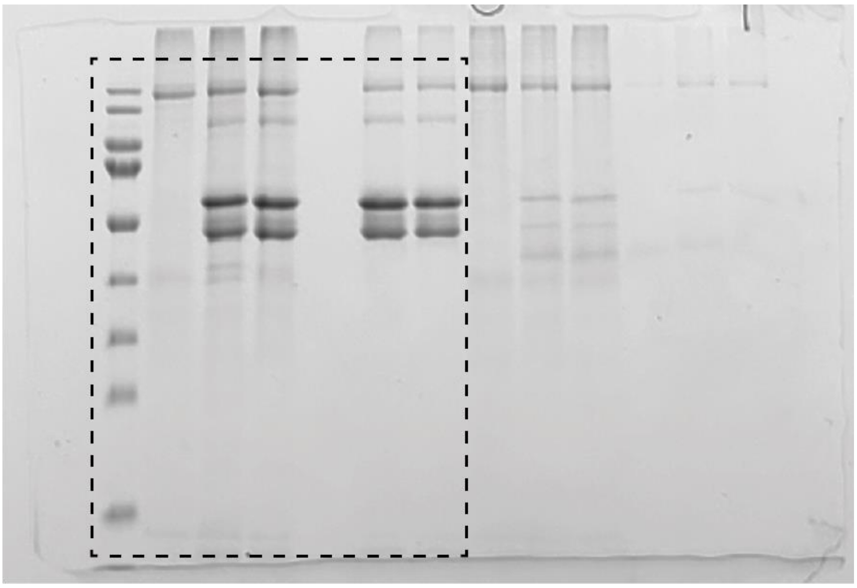

Supplement: Figure 4—source data 3. [file elife-85432-fig4-data3.zip › Figure 4-source data 3/Figure 4-source data 3.pdf]
